# Genetic Factors Associated with Increased Host Defense Antimicrobial Peptide Resistance in Sequence Type 5 Healthcare-Associated MRSA Clinical Isolates

**DOI:** 10.3390/biom10101415

**Published:** 2020-10-07

**Authors:** Kyoung-Mi Kang, Gi Yong Lee, Soo-Jin Yang

**Affiliations:** Department of Animal Science and Technology, School of Bioresources and Bioscience, Chung-Ang University, Anseong 17546, Korea; kkm1038@nate.com (K.-M.K.); dominic3809@naver.com (G.Y.L.)

**Keywords:** ST5 HA-MRSA, ST72 CA-MRSA, ST72 LA-MRSA, antimicrobial peptide resistance

## Abstract

Sequence type (ST) 72 methicillin-resistant *Staphylococcus aureus* with staphylococcal cassette chromosome *mec* (SCC*mec*) type IV (ST72-MRSA-IV) and ST5-MRSA-II are the most significant lineages found in community-associated (CA) and healthcare-associated (HA) environments in Korea, respectively. ST5 HA-MRSA-II tend to display enhanced resistance to host defense-cationic antimicrobial peptides (HD-CAPs) compared to ST72 CA-MRSA-IV and ST72 livestock-associated (LA)-MRSA-IV due to mechanisms involving a higher surface positive charge. Thus, the present study explored the genetic factors contributing to the enhanced HD-CAP resistance phenotype in ST5 MRSA strains. The ST5 HA-MRSA-II strains displayed higher levels of *mprF* and *dltABCD* expression compared to the ST72 CA-/LA-MRSA-IV strains. The increase in expression of *mprF* and *dltABCD* in ST5 HA-MRSA-II strains was correlated with dysregulation of the upstream transcriptional regulator, *graRS*. However, single nucleotide polymorphisms (SNPs) within *mprF* and *graRS* ORFs were not involved in the enhanced surface positive charge or the altered expression of *mprF*/*dltABCD*.

## 1. Introduction

Methicillin-resistant *Staphylococcus aureus* (MRSA) is a serious nosocomial pathogen that can lead to septicemia and death [1]. Besides healthcare-associated (HA)-MRSA infections, the incidence of human infections with community-associated (CA)-MRSA and livestock-associated (LA)-MRSA has also been increasing in recent years [2,3,4,5]. Clonal lineages of HA-, CA-, and LA-MRSA isolates and their distributions vary according to geographical region [4,5,6]. Sequence type (ST) 5 MRSA with staphylococcal cassette chromosome *mec* (SCC*mec*) type II (ST5-MRSA-II) and ST72-MRSA-IV represent the most significant HA- and CA-MRSA clones in Korea, respectively [6,7,8]. However, the differences in virulence factors critical for the clinical outcomes of infections caused by ST5-MRSA-II and ST72-MRSA-IV are largely unknown.

Host defense cationic antimicrobial peptides (HD-CAPs) play a critical role in host innate immune defense against bacterial infections including MRSA [9]. In a recent report from our laboratory, we showed that ST5 HA-MRSA II strains tended to be more resistant to HD-CAPs of different origins compared to ST72 CA-/LA-MRSA-IV strains [10]. In particular, ST5 HA-MRSA strains displayed higher levels of resistance to LL-37 (human cathelicidin), BMAP-28 (bovine myeloid antimicrobial peptide), and polymyxin B (bacterial antimicrobial peptide) than ST72 CA-/LA-MRSA-IV strains via mechanisms involving enhanced cell surface positive charge [10]. Over the past several years, the enhanced surface positive charge in *S. aureus* has been linked to (i) enhanced transcription of *mprF*, *dltABCD*, or both [11,12,13], (ii) single nucleotide polymorphisms (SNPs) within the *mprF* ORF (the gain-in-function mutations) [14,15,16,17], or (iii) perturbed upstream transcriptional regulation of *mprF* and *dltABCD* by the two-component regulatory system (TCRS), *graRS* [18,19,20].

Based on these prior findings, the present study aimed to define genetic factors associated with the enhanced HD-CAP resistance in ST5 HA-MRSA-II strains. Using the same 26 MRSA strains as those used in our previous study [10], we examined the transcriptional profiles of *mprF*, *dltABCD*, and *graRS* during the exponential- and stationary-growth phases. Next, sequences of *mprF* ORFs were analyzed to identify SNPs linked to the enhanced surface positive charge in ST5 HA-MRSA strains. Moreover, correlations between SNPs within *graRS* ORFs and the dysregulation of *mprF*/*dltABCD* were determined.

## 2. Materials and Methods

### 2.1. S. aureus Strains and Culture

The MRSA strains used in this investigation are listed in Table 1. We used the 26 MRSA strains previously described by Kang et al.: 8 ST5 HA-MRSA-II strains [10], 11 ST72 CA-MRSA IV strains [21], and 7 ST72 LA-MRSA IV strains [22].

All 26 MRSA strains were cultured in either Mueller–Hinton broth (MHB; Difco Laboratories, Detroit, MI, USA) or Tryptic Soy broth (TSB; Difco Laboratories) for each experiment. All MRSA cultures were incubated in 500 mL Erlenmeyer flasks at 37 °C with shaking (200 rpm) in less than 15% of the flask’s volume for optimal aeration.

### 2.2. Sequencing and Cloning of mprF and graRS

Genomic DNA samples were prepared from the MRSA strains using a method described previously [23]. For sequence analyses, the *mprF* and *graRS* ORFs were amplified through PCR using *mprF*- or *graRS*-specific primer pairs as described previously [12,20]. The PCR-amplified *mprF* and *graRS* ORFs from 26 MRSA strains were sequenced at Cosmo Genetech, Seoul, Korea. SNPs within the *mprF* and *graRS* ORFs were identified using a multiple sequence alignment tool on the BoxShade server (embnet.vital-it.ch/software/BOX_form.html).

To assess the role of the SNPs identified within *mprF* ORFs in conferring a positive charge to the cell surface, mutated or nonmutated *mprF* genes were amplified from each MRSA strain (HA7 or CA7 strains) and then expressed in the *S. aureus* Newman ∆*mprF* mutant strain [24]. Complementation of the *mprF* genes in Newman ∆*mprF* was achieved using the previously described shuttle vector, pRB474 [16,25]. Similarly, the role of SNPs identified within *graS* ORFs in the regulation of *mprF* and *dltABCD* expression was evaluated by expressing the mutated or nonmutated *graS* genes in the *S. aureus* MW2 ∆*graS* mutant strain [20]. The *graS* genes from MRSA strains with or without *graS* SNPs were PCR amplified and then ligated into pRB474 using the BamHI and SpHI sites.

Plasmid DNA was prepared from *Escherichia coli* and *S. aureus* strains using the Wizard Plus SV Miniprep kit (Promega, Madison, WI, USA). Transformation of the plasmid constructs into *E. coli* DH5α or *S. aureus* strains was accomplished as described previously [26,27].

### 2.3. Quantification of Ttranscriptional Expression by RT-qPCR

To assess the expression levels of *mprF*, *dltABCD*, and *graRS* during different growth phases, RNA samples were prepared from broth cultures of the 26 MRSA strains in the exponential and stationary growth phases. For RNA isolation, overnight cultures of the MRSA strains were used to inoculate 50 mL of fresh TSB to an OD600 of 0.05. MRSA cell pellets were then collected at either the exponential (3 h) or stationary (12 h) growth phase of the cultures. Total cellular RNA samples were prepared using the FastPrep FP120 Cell Disrupter instrument (Bio101, Vista, CA, USA) and the RNeasy kit (Qiagen, Valencia, CA, USA) as described in earlier study [28].

For quantitative real-time PCR (RT-qPCR) assays, 3 µg of each RNA sample was reverse transcribed using the Superscript III first-strand synthesis system (Thermo Fisher Scientific), as recommended by the manufacturer. Quantification of *mprF*, *dltA*, and *graRS* cDNA levels was carried out according to the protocol described by the manufacturer of the Power SYBR green master mix kit in a LineGene 9600 Plus system (Bioer Technology, Hangzhou, China). The transcripts of *mprF*, *dltABCD*, *graRS*, and *gyrB* were amplified using their respective gene-specific primers, as explained previously [18,29,30]. Fold changes in the transcription levels of all genes were quantified in relation to the transcription level of *gyrB*. At least two independent experiments were carried out for each RNA sample

### 2.4. Net Cell Surface Charge in MRSA Strains

The binding of cytochrome *c* (Sigma-Aldrich, St. Louis, MO, USA) to the MRSA cell surface was measured to determine the relative surface positive charge using the spectrophotometric method as described in prior publications [16,31]. Three independent cytochrome *c* binding assays were carried out on separate days.

### 2.5. Statistical Analyses

The Kruskal–Wallis ANOVA test with the Tukey post hoc correlation was performed for multiple comparisons (IBM SPSS Statistics 23, Chicago, IL, USA). Statistical significance was considered at *p* < 0.05.

## 3. Results

### 3.1. SNPs within mprF and graRS ORFs

All 26 MRSA strains were subjected to sequencing analyses for *mprF* and *graRS* genes. As presented in Table 1, sequencing analyses of the *mprF* ORFs revealed that all the 18 ST72 MRSA-IV strains (7 ST72 LA-MRSA-IV and 11 ST72 CA-MRSA-IV strains) had two nonsynonymous mutations (ATT→ATG at position 1125; ATT→ACT at position 1382) within *mprF* ORFs distinct from those of the ST5 HA-MRSA-II strains, resulting in I375M and I461T substitutions, respectively (I375M, isoleucine to methionine at position 375; I461T, isoleucine to threonine at position 461). Similarly, all of the eight ST5 HA-MRSA-II strains exhibited a distinctive I224T amino acid substitution in the *graS* ORF compared to the 18 ST72 MRSA-IV strains. In addition to the two most common *mprF* mutations in ST72 MRSA strains (I375M and I461T), the amino acid substitutions F550C and T666I were identified in two ST72 MRSA-IV strains (LA2 and CA1 strains). Although a few MRSA strains had amino acid substitutions in *graR* ORF, such as K100N, E15K, and V87G, the mutations resulting in such substitutions were observed in only one strain each of ST5 HA-MRSA and ST72 CA-MRSA.

### 3.2. Effect of mprF SNPs on The Net Cell Surface Positive Charge

A number of previous publications have demonstrated that the presence of SNPs within the mprF ORF is often correlated with an increase in net cell surface positive charge as a result of either enhanced synthesis or outer membrane translocation of lysyl-phosphatidylglycerol (L-PG) in *S. aureus* [12,13,17,32,33,34,35].

**Figure 1:**
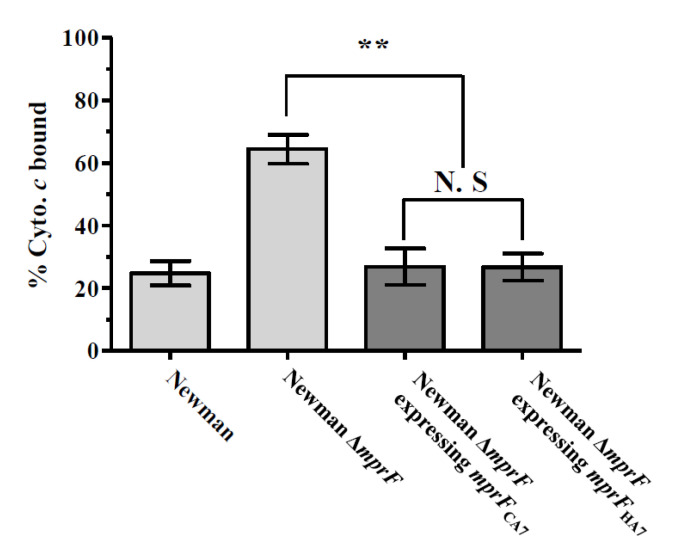
Effect of *mprF* SNPs (I375M and I461T) on the net surface positive charge in *Staphylococcus aureus.* The graph shows the percentage of positively-charged cytochrome *c* bound after 15 min of incubation with each *S. aureus* strain at room temperature. ** *p* < 0.01; N.S, not significant.

To determine whether the two amino acid substitutions (I375M and I461T) observed in ST72 MRSA strains contribute to the cell surface positive charge phenotype, Newman ∆mprF strains with plasmids expressing one of two different forms of mprF genes (mprF_HA7_ or mprF_CA7_) were subjected to cytochrome c binding analysis. As shown in Figure 1, the two ∆mprF strains expressing mprF genes either with or without the I375M and I461T substitutions did not show any difference in bacterial cell surface charge, indicating that these two amino acid substitutions in the ST72 MRSA strains are not involved in surface charge regulation in *S. aureus*.

### 3.3. Expression of mprF, dltA, and graS in MRSA Strains

**Figure 2:**
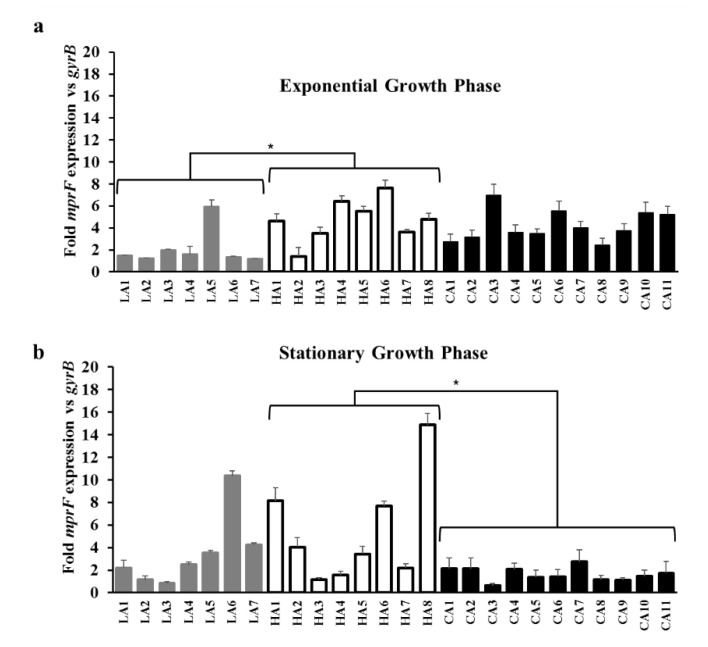
Transcriptional expression of *mprF* in the MRSA strains during the exponential (**a**) and stationary (**b**) growth phases. RNA samples from the 26 MRSA strains cultured in Mueller–Hinton broth were isolated 3 h (exponential growth phase) and 12 h (stationary growth phase) after initial inoculation and used for RT-qPCR assays. The fold expression of *mprF* was quantified relative to the levels of *gyrB* expression. * *p* < 0.05.

**Figure 3:**
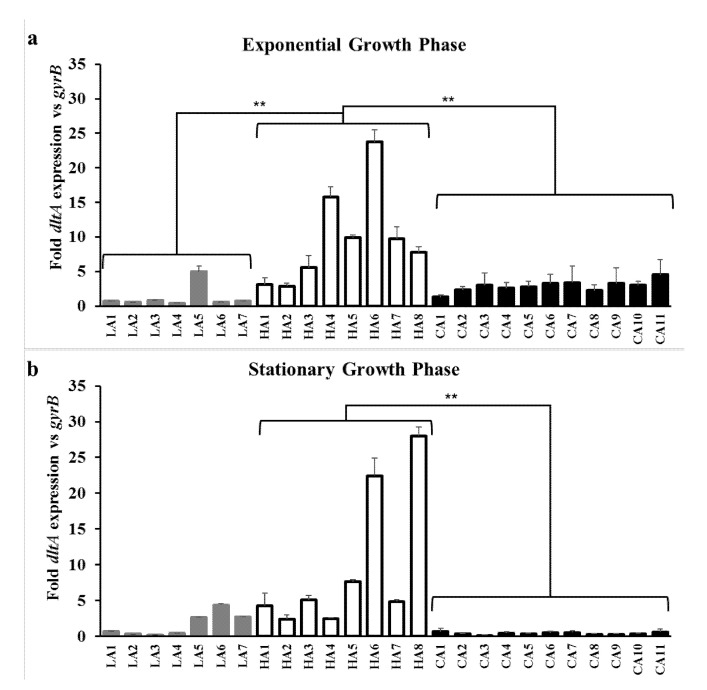
Transcriptional expression of *dltA* in the MRSA strains during the exponential (**a**) and stationary (**b**) growth phases. RNA samples from the 26 MRSA strains cultured in Mueller–Hinton broth were isolated 3 h (exponential growth phase) and 12 h (stationary growth phase) after initial inoculation and used for RT-qPCR assays. The fold expression of *dltA* was quantified relative to the levels of *gyrB* expression. ** *p* < 0.01.

As shown in Figure 2a,b, RT-qPCR analyses revealed that ST5 HA-MRSA strains had significantly higher levels of *mprF* transcription than the ST72 CA-MRSA and ST72 LA-MRSA strains either in the exponential or stationary growth phase (*p* < 0.05). Moreover, since *dltABCD* genes also contribute to the net surface positive charge in *S. aureus* [12,29], the transcriptional profiles of *dltA* were determined in the 26 MRSA strains assessed herein. As presented in Figure 3a,b, ST5 HA-MRSA strains exhibited significantly enhanced *dltA* expression levels during the exponential growth phase compared to the ST72 LA-MRSA and ST72 CA-MRSA strains (*p* < 0.01). The difference in *dltA* expression between the ST5 HA-MRSA and ST72 CA-MRSA strains continued to the stationary growth phase (*p* < 0.01) (Table 2).

**Figure 4:**
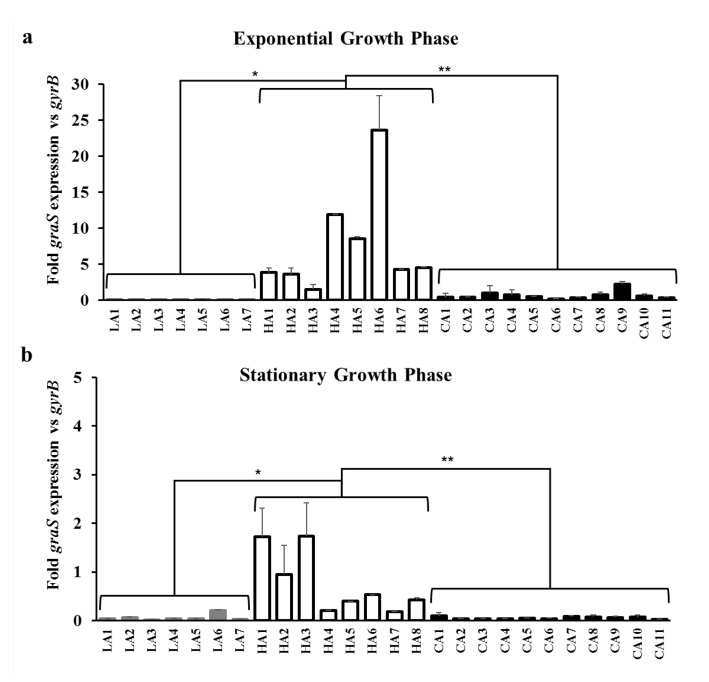
Transcriptional expression of *graS* in the MRSA strains during the exponential (**a**) and stationary (**b**) growth phases. RNA samples from the 26 MRSA strains cultured in Mueller–Hinton broth were isolated 3 h (exponential growth phase) and 12 h (stationary growth phase) after initial inoculation and used for RT-qPCR assays. The fold expression of *graS* was quantified relative to the levels of *gyrB* expression. * *p* < 0.05; ** *p* < 0.01.

Since the ST5 HA-MRSA strains tended to transcribe higher levels of *mprF* and *dltA* than the ST72 LA-/CA-MRSA strains, the expression levels of *graRS*, the transcriptional regulator of *mprF* and *dltABCD* [19], were determined in the 26 MRSA strains. The ST5 HA-MRSA strains exhibited significantly higher expression levels of *graS* during both exponential and stationary growth phases compared to the two ST72 MRSA strain groups (Figure 4a,b).

### 3.4. Effect of GraS SNPs on the Regulation of mprF and dltABCD Transcription

**Figure 5:**
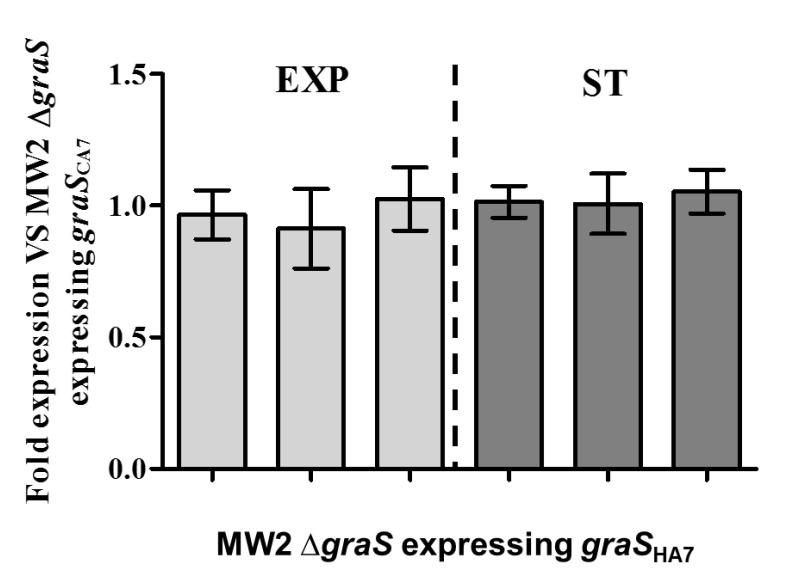
Effect of *graS* SNP (I224T) on the transcription of *graS*, *mprF*, and *dltA* in *S. aureus* during the exponential and stationary growth phases. RNA samples from the two MW2 ∆*graS* strains expressing *graRS* genes cloned either from CA7 or HA7 strains were isolated 3 h post-inoculation. Fold changes in the expression levels of *graS*, *mprF*, and *dltA* in MW2 ∆*graS*_HA7_ were normalized to the levels of the expression of their respective target genes in MW2 ∆*graS*_CA7_.

The prototypical two-component regulatory system (TCRS) GraRS up-regulates the transcription of *mprF* and *dltABCD*, which encode protein products that modify the net surface charge in *S. aureus* [11,18,20,36,37]. To assess whether the amino acid substitution I224T within *graS* genes in ST5 HA-MRSA strains affects the expression of downstream target genes such as *mprF* and *dltA*, *S. aureus* MW2 ∆*graS* strains expressing either *graS*_CA7_ or *graS*_HA7_ in trans were subjected to RT-qPCR analysis. As presented in Figure 5, the two strains displayed almost identical levels of *graS*, *mprF*, and *dltA* transcription, suggesting that I224T substitution is not involved in the dysregulation of *graS*, *mprF*, and *dltA* in ST5 HA-MRSA strains.

## 4. Discussion

Previously, we have shown that ST5 HA-MRSA-II isolates, the most significant HA-MRSA in Korea, were more resistant to HD-CAPs of human, bovine, and prokaryotic origins compared with human- and animal-derived ST72 MRSA-IV isolates [10]. The observed enhancement in resistance to bactericidal action of HD-CAP in ST5 HA-MRSA was associated with an enhanced surface positive charge [10]. Thus, the current study was designed to explore the genetic factors responsible for the enhanced surface positive charge in ST5 HA-MRSA strains compared to those in ST72 CA-/LA-MRSA-IV strains.

Sequencing analyses of *mprF* ORFs in the 26 MRSA strains revealed that there are genotype specific SNPs within these gene loci (Table 1). Previous studies have demonstrated that SNPs within specific domains of MprF are often associated with increased synthesis or translocation of positively charged L-PG, and thus contributes to HD-CAP resistance via charge repulsion mechanisms [32,34,35,38]. The two *mprF* SNPs (I375M and I461T) identified in the ST72 CA-/LA-MRSA strains in this study were reported in a previous publication in a daptomycin-nonsusceptible (DAP-NS) ST72 MRSA strain that displayed an enhanced surface positive charge versus DAP-susceptible MRSA strains [39]. However, in a recent publication [10], the 18 ST72 MRSA-IV strains with the I375M and I461T mutations were shown to have reduced levels of surface positive charge compared to those of the ST5 HA-MRSA strains. Therefore, to determine whether the I375M and I461T mutations in the *mprF* ORF are directly linked to the enhanced surface positive charge, and thus the HD-CAP resistance phenotype of ST5 HA-MRSA strains, the Newman ∆*mprF* strains expressing the *mprF* genes from ST72 MRSA (*mprF*I375M+I461T) or ST5 MRSA (*mprF*) strains were subjected to cytochrome *c* binding assays. As presented in Figure 1, complementation of the Newman ∆*mprF* strain with *mprF*I375M+I461T did not result in an enhanced surface positive charge versus the Newman ∆*mprF* strain expressing a nonmutated form of *mprF*, suggesting that the two SNPs (I375M and I461T) are likely not involved in the previously observed increase in the surface positive charge of the ST5 HA-MRSA II strains [10,39]. Bayer et al. also reported frequent SNPs within *mprF* ORFs among clinical DAP-NS MRSA strains, but only a limited number of SNPs within the hot spot loci of *mprF* were shown to be linked to the gain-of-function phenotype that resulted in enhanced surface positive charge [40]. These results indicate that the heterogeneity within *mprF* ORFs is not associated with the higher levels of surface positive charge and enhanced resistance to HD-CAP in the ST5 HA-MRSA-II strains compared to those of the ST72 CA-/LA-MRSA-IV strains.

Besides the gain-of-function mutations in *mprF* ORFs [12,13,14,17,32,34,35], it has been previously reported that the increased transcription of *mprF*, *dltABCD*, or both, resulted in altered surface positive charge in DAP-NS and HD-CAP resistant *S. aureus* [11,36]. Along with MprF, *dltABCD* gene products contribute to the accumulation of net surface positive charge in *S. aureus* by increasing D-alanylation of teichoic acids in the peptidoglycan cell wall [12,41]. The *graRS* TCRS also contributes to surface positive charge regulation in staphylococci by inducing the transcription of target genes, such as *mprF*, *dltABCD*, and *vraFG* [11,18,36,37]. As shown in Figure 2 and Figure 3 and Table 2, ST5 HA-MRSA-II strains displayed significantly higher levels of *mprF* and *dltA* expression during exponential and stationary growth phases compared with those of ST72 CA-/LA-MRSA-II strains. In combination with previous results [10], data obtained in the present study suggest that the higher level of HD-CAP resistance in ST5 HA-MRSA-II compared to that in ST72 MRSA strains due to enhanced surface positive charge is correlated with the increased transcription of *mprF* and *dltABCD*. Since similar overall profiles of *mprF* and *dltA* transcription in the 26 MRSA strains were observed (Figure 2 and Figure 3), the co-regulation of these two genetic factors by the upstream transcriptional regulator *graRS* was determined. As presented in Figure 4 and Table 2, the ST5 HA-MRSA-II strains exhibited a significantly enhanced transcription of *graS* throughout the two growth phases, correlating well with the enhanced expression of *mprF* and *dltABCD* in ST5 HA-MRSA-II strains compared to that in the two groups of ST72 MRSA-IV strains. Furthermore, as shown in Table 1, sequencing analyses of *graRS* ORFs revealed that all ST5 HA-MRSA-II strains carried a SNP in the *graS* ORF, which caused an amino acid substitution of I224T distinct from those in ST72 MRSA strains. However, complementation of the MW2 ∆*graS* strain either with *graS* from the CA7 or HA7 strains failed to show any difference in *mprF*, *dltA*, and *graS* expression (Figure 5), suggesting that the I224T mutation in ST5 HA-MRSA strains was not involved in the dysregulation of *mprF* and *dltABCD* expression. Sequence analyses of *graRS* promoter regions in the HA1, HA6, LA1, and CA1 strains also revealed that all the four strains had identical promoter sequences (data not shown), demonstrating that the dysregulation of *graS* expression in ST5 HA-MRSA-II strains was not resulted from mutations in their promoter sequences.

It should also be noted that *S. aureus* generally depends on three major events to modulate its relatively more positive in charge surface: (i) _D_-alanylation of wall teichoic acids via the *dltABCD* genes, (ii) amidation of glutamine residues in the peptidoglycan cell wall via the *murT* and *gatD* genes (Munch et al., PLoS Pathogen, 2012; Figueiredo, PLoS Pathogen, 2112), and (iii) lysinylation of the PG to L-PG via the mprF in the cell membrane. In addition, a certain level of the N-acetylmuramic acid in the cell wall becomes O-acetylated on position C_6_ by the *oatA* gene product, which could affect hydrophobicity and surface charge in *S. aureus*.

## 5. Conclusions

To conclude, in combination with previously published data [10], which demonstrated that the ST5 HA-MRSA isolates were more resistant to HD-CAPs of various origins than ST72 CA-MRSA isolates via increased positive charge in cell envelopes, our current data suggest that (i) the enhanced HD-CAP resistance resulting from the enhanced net surface positive charge in ST5 HA-MRSA-II strains compared to that in the ST72 CA-/LA-MRSA-II strains was associated with the dysregulation of *mprF* and *dltABCD* expression, (ii) the altered expression of *mprF* and *dltABCD* in ST5 HA-MRSA-II strains was caused by the dysregulation of the upstream transcriptional regulator *graRS* TCRS, (iii) the two *mprF* SNPs (I375M and I461T) identified in ST72-MRSA strains did not affect net surface positive charge, and (iv) the I224T mutation in the *graS* ORF was not involved in the dysregulation of *mprF* and *dltABCD* by altered *graRS* expression in ST5 HA-MRSA-II strains.

## Figures and Tables

**Table 1:** Genotypes and SNPs in *mprF* and *graRS* ORFs of 26 MRSA strains.

			*mprF* SNPs ^a^			*graS* SNPs ^a^			*graR* SNPs ^a^		
Strain	MLST	SCC *mec*	Codon Change	^b^ Nucleotide Position	Amino Acid Change	Codon Change	^b^ Nucleotide Position	Amino Acid Change	Codon Change	^b^ Nucleotide Position	Amino Acid Change
LA1	ST72	IV	ATT→ATG ATT→ACT	1125 1382	I375M I461T	No change	^c^ NA	NA	No change	NA	NA
LA2	ST72	IV	ATT→ATG ATT→ACT TTC→TGC	1125 1382 1649	I375M I461T F550C	No change	NA	NA	No change	NA	NA
LA3	ST72	IV	ATT→ATG ATT→ACT	1125 1382	I375M I461T	No change	NA	NA	No change	NA	NA
LA4	ST72	IV	ATT→ATG ATT→ACT	1125 1382	I375M I461T	No change	NA	NA	No change	NA	NA
LA5	ST72	IV	ATT→ATG ATT→ACT	1125 1382	I375M I461T	No change	NA	NA	No change	NA	NA
LA6	ST72	IV	ATT→ATG ATT→ACT	1125 1382	I375M I461T	No change	NA	NA	No change	NA	NA
LA7	ST72	IV	ATT→ATG ATT→ACT	1125 1382	I375M I461T	No change	NA	NA	No change	NA	NA
HA1	ST5	II	No change	NA	NA	ATA→ACA	671	I224T	AAA→AAT	300	K100N
HA2	ST5	II	No change	NA	NA	ATA→ACA	671	I224T	No change	NA	NA
HA3	ST5	II	No change	NA	NA	ATA→ACA	671	I224T	No change	NA	NA
HA4	ST5	II	No change	NA	NA	ATA→ACA	671	I224T	No change	NA	NA
HA5	ST5	II	No change	NA	NA	ATA→ACA	671	I224T	No change	NA	NA
HA6	ST5	II	CAT→CCT GAG→AAG	362 802	H121P E268K	ATA→ACA	671	I224T	No change	NA	NA
HA7	ST5	II	No change	NA	NA	ATA→ACA	671	I224T	No change	NA	NA
HA8	ST5	II	No change	NA	NA	ATA→ACA	671	I224T	No change	NA	NA
CA1	ST72	IV	ATT→ATG ATT→ACT ACT→ATT	1125 1382 1997	I375M I461T T666I	No change	NA	NA	No change	NA	NA
CA2	ST72	IV	ATT→ATG ATT→ACT	1125 1382	I375M I461T	No change	NA	NA	No change	NA	NA
CA3	ST72	IV	ATT→ATG ATT→ACT	1125 1382	I375M I461T	No change	NA	NA	No change	NA	NA
CA4	ST72	IV	ATT→ATG ATT→ACT	1125 1382	I375M I461T	GAT→AAT	631	D211N	No change	NA	NA
CA5	ST72	IV	ATT→ATG ATT→ACT	1125 1382	I375M I461T	No change	NA	NA	No change	NA	NA
CA6	ST72	IV	ATT→ATG ATT→ACT	1125 1382	I375M I461T	No change	NA	NA	No change	NA	NA
CA7	ST72	IV	ATT→ATG ATT→ACT	1125 1382	I375M I461T	No change	NA	NA	No change	NA	NA
CA8	ST72	IV	ATT→ATG ATT→ACT	1125 1382	I375M I461T	No change	NA	NA	No change	NA	NA
CA9	ST72	IV	ATT→ATG ATT→ACT	1125 1382	I375M I461T	No change	NA	NA	No change	NA	NA
CA10	ST72	IV	ATT→ATG ATT→ACT	1125 1382	I375M I461T	No change	NA	NA	GTG→GGG	260	V87G
CA11	ST72	IV	ATT→ATG ATT→ACT	1125 1382	I375M I461T	No change	NA	NA	No change	NA	NA

^a^ The *mprF* and *graRS* sequences from the *S. aureus* MW2 strain were used as the consensus reference sequences to identify single nucleotide polymorphisms (SNPs) among the study strains.; ^b^ Position of nucleotide change within the *mprF* or *graRS* ORF; ^c^ NA, not applicable.

**Table 2:** Group comparison for transcriptional expression of *mprF*, *dltA*, and *graS* among LA-MRSA, HA-MRSA, and CA-MRSA strains.

Parameter	Groups			*p* Value for	
	ST72 LA-MRSA	ST5 HA-MRSA	ST72 CA-MRSA	ST72 LA-MRSA Vs. ST5 HA-MRSA	ST72 CA-MRSA Vs. ST5 HA-MRSA
Fold *mprF* expression vs. *gyrB* in:					
					
Exponential growth phase	2.12 ± 1.07	4.68 ± 1.92	4.21 ± 1.40	<0.05	^a^ NS
Stationary growth phase	3.54 ± 3.23	5.38 ± 4.64	1.63 ± 0.60	^a^ NS	<0.05
					
Fold *dltA* expression vs. *gyrB* in:					
					
Exponential growth phase	1.24 ± 1.65	9.80 ± 7.02	2.88 ± 0.81	<0.01	<0.01
Stationary growth phase	1.60 ± 1.63	9.65 ± 9.86	0.39 ± 0.16	NS	<0.01
					
Fold *graS* expression vs. *gyrB* in:					
					
Exponential growth phase	0.03 ± 0.04	7.71 ± 7.22	0.67 ± 0.55	<0.05	<0.01
Stationary growth phase	0.06 ± 0.07	0.77 ± 0.64	0.05 ± 0.02	<0.05	<0.01

^a^ NS, not significant.

## References

[1] Graffunder E.M., Venezia R.A. (2002). Risk factors associated with nosocomial methicillin-resistant Staphylococcus aureus (MRSA) infection including previous use of antimicrobials. J. Antimicrob. Chemother..

[2] Soo Ko K., Kim Y.S., Song J.H., Yeom J.S., Lee H., Jung S.I., Jeong D.R., Kim S.W., Chang H.H., Ki H.K. (2005). Genotypic diversity of methicillin-resistant Staphylococcus aureus isolates in Korean hospitals. Antimicrob. Agents Chemother..

[3] Okuma K., Iwakawa K., Turnidge J.D., Grubb W.B., Bell J.M., O’Brien F.G., Coombs G.W., Pearman J.W., Tenover F.C., Kapi M. (2002). Dissemination of new methicillin-resistant Staphylococcus aureus clones in the community. J. Clin. Microbiol..

[4] Mediavilla J.R., Chen L., Mathema B., Kreiswirth B.N. (2012). Global epidemiology of community-associated methicillin resistant Staphylococcus aureus (CA-MRSA). Curr. Opin. Microbiol..

[5] Price L.B., Stegger M., Hasman H., Aziz M., Larsen J., Andersen P.S., Pearson T., Waters A.E., Foster J.T., Schupp J. (2012). Staphylococcus aureus CC398: Host adaptation and emergence of methicillin resistance in livestock. mBio.

[6] Song J.-H., Hsueh P.-R., Chung D.R., Ko K.S., Kang C.-I., Peck K.R., Yeom J.-S., Kim S.-W., Chang H.-H., Kim Y.-S. (2011). Spread of methicillin-resistant Staphylococcus aureus between the community and the hospitals in Asian countries: An ANSORP study. J. Antimicrob. Chemother..

[7] Kim E.S., Song J.S., Lee H.J., Choe P.G., Park K.H., Cho J.H., Park W.B., Kim S.-H., Bang J.-H., Kim D.-M. (2007). A survey of community-associated methicillin-resistant Staphylococcus aureus in Korea. J. Antimicrob. Chemother..

[8] Joo E.-J., Chung D., Ha Y., Park S., Kang S.-J., Kim S., Kang C.-I., Peck K., Lee N., Ko K. (2012). Community-associated Panton–Valentine leukocidin-negative meticillin-resistant Staphylococcus aureus clone (ST72-MRSA-IV) causing healthcare-associated pneumonia and surgical site infection in Korea. J. Hosp. Infect..

[9] Mishra N.N., Bayer A.S., Moise P.A., Yeaman M.R., Sakoulas G. (2012). Reduced susceptibility to host-defense cationic peptides and daptomycin coemerge in methicillin-resistant Staphylococcus aureus from daptomycin-naive bacteremic patients. J. Infect. Dis..

[10] Kang K.M., Park J.H., Kim S.H., Yang S.J. (2019). Potential role of host defense antimicrobial peptide resistance in increased virulence of health care-associated MRSA strains of sequence type (ST) 5 versus livestock-associated and community-associated MRSA strains of ST72. Comp. Immunol. Microbiol. Infect. Dis..

[11] Mishra N.N., Yang S.-J., Sawa A., Rubio A., Nast C.C., Yeaman M.R., Bayer A.S. (2009). Analysis of cell membrane characteristics of in vitro-selected daptomycin-resistant strains of methicillin-resistant Staphylococcus aureus. Antimicrob. Agents Chemother..

[12] Yang S.J., Kreiswirth B.N., Sakoulas G., Yeaman M.R., Xiong Y.Q., Sawa A., Bayer A.S. (2009). Enhanced expression of dltABCD is associated with the development of daptomycin nonsusceptibility in a clinical endocarditis isolate of Staphylococcus aureus. J. Infect. Dis..

[13] Yang S.J., Xiong Y.Q., Dunman P.M., Schrenzel J., Francois P., Peschel A., Bayer A.S. (2009). Regulation of mprF in daptomycin-nonsusceptible Staphylococcus aureus strains. Antimicrob. Agents Chemother..

[14] Mishra N.N., Yang S.J., Chen L., Muller C., Saleh-Mghir A., Kuhn S., Peschel A., Yeaman M.R., Nast C.C., Kreiswirth B.N. (2013). Emergence of daptomycin resistance in daptomycin-naive rabbits with methicillin-resistant Staphylococcus aureus prosthetic joint infection is associated with resistance to host defense cationic peptides and mprF polymorphisms. PLoS ONE.

[15] Yang S.J., Mishra N.N., Kang K.M., Lee G.Y., Park J.H., Bayer A.S. (2018). Impact of multiple single-nucleotide polymorphisms within mprF on daptomycin resistance in Staphylococcus aureus. Microb. Drug Resist..

[16] Yang S.-J., Mishra N.N., Rubio A., Bayer A.S. (2013). Causal role of single nucleotide polymorphisms within the mprF gene of Staphylococcus aureus in daptomycin resistance. Antimicrob. Agents Chemother..

[17] Bayer A.S., Mishra N.N., Chen L., Kreiswirth B.N., Rubio A., Yang S.J. (2015). Frequency and distribution of single-nucleotide polymorphisms within mprF in methicillin-resistant Staphylococcus aureus clinical isolates and their role in cross-resistance to daptomycin and host defense antimicrobial peptides. Antimicrob. Agents Chemother..

[18] Yang S.J., Bayer A.S., Mishra N.N., Meehl M., Ledala N., Yeaman M.R., Xiong Y.Q., Cheung A.L. (2012). The Staphylococcus aureus two-component regulatory system, GraRS, senses and confers resistance to selected cationic antimicrobial peptides. Infect Immun..

[19] Chaili S., Cheung A.L., Bayer A.S., Xiong Y.Q., Waring A.J., Memmi G., Donegan N., Yang S.J., Yeaman M.R. (2016). The GraS sensor in Staphylococcus aureus mediates resistance to host defense peptides differing in mechanisms of action. Infect Immun..

[20] Cheung A.L., Bayer A.S., Yeaman M.R., Xiong Y.Q., Waring A.J., Memmi G., Donegan N., Chaili S., Yang S.J. (2014). Site-specific mutation of the sensor kinase GraS in Staphylococcus aureus alters the adaptive response to distinct cationic antimicrobial peptides. Infect Immun..

[21] Kim E.S., Bae I.-G., Cho J.E., Choi Y.J., Kim I.-H., Kang G.-S., Sin H.-y., Song K.-H., Park C., Lee D.-G. (2016). Clinical and molecular characterization of invasive heteroresistant vancomycin-intermediate Staphylococcus aureus infections in Korean hospitals. J. Clin. Microbiol..

[22] Song J.W., Yang S.J., Shin S., Seo K.S., Park Y.H., Park K.T. (2016). Genotypic and phenotypic characterization of methicillin-resistant Staphylococcus aureus isolated from bovine mastitic milk in Korea. J. Food Prot..

[23] Dyer D.W., Iandolo J.J. (1983). Rapid isolation of DNA from Staphylococcus aureus. Appl. Environ. Microbiol..

[24] Peschel A., Jack R.W., Otto M., Collins L.V., Staubitz P., Nicholson G., Kalbacher H., Nieuwenhuizen W.F., Jung G., Tarkowski A. (2001). Staphylococcus aureus resistance to human defensins and evasion of neutrophil killing via the novel virulence factor MprF is based on modification of membrane lipids with l-lysine. J. Exp. Med..

[25] Bruckner R. (1992). A series of shuttle vectors for Bacillus subtilis and Escherichia coli. Gene.

[26] Inoue H., Nojima H., Okayama H. (1990). High efficiency transformation of Escherichia coli with plasmids. Gene.

[27] Schenk S., Laddaga R.A. (1992). Improved method for electroporation of Staphylococcus aureus. FEMS Microbiol. Lett..

[28] Lan L., Cheng A., Dunman P.M., Missiakas D., He C. (2010). Golden pigment production and virulence gene expression are affected by metabolisms in Staphylococcus aureus. J. Bacteriol..

[29] Bertsche U., Weidenmaier C., Kuehner D., Yang S.J., Baur S., Wanner S., Francois P., Schrenzel J., Yeaman M.R., Bayer A.S. (2011). Correlation of daptomycin resistance in a clinical Staphylococcus aureus strain with increased cell wall teichoic acid production and D-alanylation. Antimicrob. Agents Chemother..

[30] Bayer A.S., Mishra N.N., Cheung A.L., Rubio A., Yang S.J. (2016). Dysregulation of mprF and dltABCD expression among daptomycin-non-susceptible MRSA clinical isolates. J. Antimicrob. Chemother..

[31] Yang S.J., Nast C.C., Mishra N.N., Yeaman M.R., Fey P.D., Bayer A.S. (2010). Cell wall thickening is not a universal accompaniment of the daptomycin nonsusceptibility phenotype in Staphylococcus aureus: Evidence for multiple resistance mechanisms. Antimicrob. Agents Chemother..

[32] Jones T., Yeaman M.R., Sakoulas G., Yang S.J., Proctor R.A., Sahl H.G., Schrenzel J., Xiong Y.Q., Bayer A.S. (2008). Failures in clinical treatment of Staphylococcus aureus infection with daptomycin are associated with alterations in surface charge, membrane phospholipid asymmetry, and drug binding. Antimicrob. Agents Chemother..

[33] Murthy M.H., Olson M.E., Wickert R.W., Fey P.D., Jalali Z. (2008). Daptomycin non-susceptible meticillin-resistant Staphylococcus aureus USA 300 isolate. J. Med. Microbiol..

[34] Ernst C.M., Staubitz P., Mishra N.N., Yang S.J., Hornig G., Kalbacher H., Bayer A.S., Kraus D., Peschel A. (2009). The bacterial defensin resistance protein MprF consists of separable domains for lipid lysinylation and antimicrobial peptide repulsion. PLoS Pathog..

[35] Ernst C.M., Kuhn S., Slavetinsky C.J., Krismer B., Heilbronner S., Gekeler C., Kraus D., Wagner S., Peschel A. (2015). The lipid-modifying multiple peptide resistance factor is an oligomer consisting of distinct interacting synthase and flippase subunits. mBio.

[36] Peschel A., Otto M., Jack R.W., Kalbacher H., Jung G., Götz F. (1999). Inactivation of the dlt operon in Staphylococcus aureus confers sensitivity to defensins, protegrins, and other antimicrobial peptides. J. Biol. Chem..

[37] Meehl M., Herbert S., Gotz F., Cheung A. (2007). Interaction of the GraRS two-component system with the VraFG ABC transporter to support vancomycin-intermediate resistance in Staphylococcus aureus. Antimicrob. Agents Chemother..

[38] Mishra N.N., Bayer A.S., Tran T.T., Shamoo Y., Mileykovskaya E., Dowhan W., Guan Z., Arias C.A. (2012). Daptomycin resistance in enterococci is associated with distinct alterations of cell membrane phospholipid content. PLoS ONE.

[39] Nam E.Y., Yang S.J., Kim E.S., Cho J.E., Park K.H., Jung S.I., Yoon N., Kim D.M., Lee C.S., Jang H.C. (2018). Emergence of daptomycin-nonsusceptible methicillin-resistant Staphylococcus aureus clinical isolates among daptomycin-naive patients in Korea. Microb. Drug Resist..

[40] Bayer A.S., Mishra N.N., Sakoulas G., Nonejuie P., Nast C.C., Pogliano J., Chen K.T., Ellison S.N., Yeaman M.R., Yang S.J. (2014). Heterogeneity of mprF sequences in methicillin-resistant Staphylococcus aureus clinical isolates: Role in cross-resistance between daptomycin and host defense antimicrobial peptides. Antimicrob. Agents Chemother..

[41] Mishra N.N., Bayer A.S., Weidenmaier C., Grau T., Wanner S., Stefani S., Cafiso V., Bertuccio T., Yeaman M.R., Nast C.C. (2014). Phenotypic and genotypic characterization of daptomycin-resistant methicillin-resistant Staphylococcus aureus strains: Relative roles of mprF and dlt operons. PLoS ONE.

